# News and Coming Events

**Published:** 1907-06-29

**Authors:** 


### NEWS AND COMING EVENTS.

Mr. McAdam Eccles, M.S., F.R.C.S., has consented to deliver the next Lees and Raper Memorial Lecture.

Dr. Kelynack, M.D., M.R.C.P., has been appointed Honorary Physician and Medical Adviser to the Children's Home and Orphanage (headquarters Bonner Road, N.E., and nine country branches.)

The July issue of the *Journal of Tuberculosis* will be a special number dealing with the tuberculosis problem as it concerns infants and children, a department which hitherto has been too long neglected by British hygienists.

At the next meeting of the Society for the Study of Inebriety on Tuesday, July 9, at 4 p.m., in the rooms of the Medical Society of London, 11 Chandos Street, Cavendish Square, W., Mrs. Mary Scharlieb, M.D., M.S., will open a discussion on "Alcohol and the Children of the Nation."

The Eleventh International Congress against Alcoholism will be held at Stockholm from July 28 to August 3 next. H.R.H. Prince Gustaf Adolf has consented to become Hon. President of the Congress. The opening oration will be delivered by Professor Tigerstedt, of Helsingfors University, and an important provisional programme has been arranged. Particulars about the Congress may be obtained on application to the Congress Bureau, Tignergatan No. 2 Stockholm.

The third reading of King Edward's Hospital Fund for London Bill has been blocked by more than one member. Dr. Rutherford has given notice that he will move the recommittal of this Bill, with instructions to the Committee to amend it by vesting the control and management of the Fund in the President and Council, the latter being constituted on representative lines. This motion is set down for June 27, after we go to press.

On the 18th inst. Lord Armstrong opened the sanatorium, which has been erected at Barrasford by the Newcastle and Northumberland branch of the National Association for the Prevention of Consumption and other forms of Tuberculosis. An excellent site has been selected. Before the opening ceremony took place there was a debt of £3,100 on the buildings. This was substantially reduced before the termination of the proceedings; but, even when the deficit is wiped out, there is still great need for increased public support, in order that the accommodation may be increased and that the charge made to patients may be reduced.

At the seventh annual general meeting of the shareholders of Virol, Limited, Mr. B. S. Straus, M.P., referred to the high rate of infant mortality in the country, and quoted from a statistical study of the question by Dr. Newsholme, Medical Officer of Health for Brighton, that out of every 1,000 born in England and Wales during the years 1901-1904, 141 died before reaching their first anniversary, and that this rate of infant mortality had remained stationary during the last fifty years, although the death-rate between the ages of five and twenty-five had been reduced by 50 per cent. in the same time. During the same period the birth-rate has fallen 17 per cent. The question of the saving of infant life was therefore a matter of imperial moment. The causes of death enumerated by the Registrar-General as accountable for this appalling rate of mortality were the very conditions that the use of Virol as a food, is claimed to obviate.

The annual distribution of prizes to the students of the St. Mary's Hospital Medical School will take place on Thursday afternoon, July 11th, at 3.30 o'clock. The prizes will be presented by Professor Osler, M.D., F.R.S.

Mr. W. J. Morton is to be congratulated upon the success of his great efforts to place the affairs of the Mount Vernon Hospital for Consumption at Hampstead and Northwood upon a sound financial basis. Last year when legacies were taken into account, the over expenditure only amounted to £700. At the festival dinner on the 24th inst., despite the extraordinary depression which has blighted the City of London for several years past, Mr. Morton succeeded in raising no less a sum than £3,650. In all the circumstances we consider such a result constitutes a feat of which Mr. Morton may well be proud.

Under the will of Mrs. Stephanie Roper, of Cricklewood House, Elsworthy Road, Hampstead, formerly in business as a Court dressmaker, and a well-known philanthropist in North London, about £16,000 has been bequeathed for charitable purposes. The testatrix left estate valued at £31,466, and made the following charitable bequests to hospitals: Middlesex Hospital, £1,050; Charing Cross Hospital, £1,000; North London Hospital for Consumption, Mount Vernon, £1,000; Victoria Hospital for Children, Chelsea, £1,000; Guy's Hospital, £1,000; St. Mary's Hospital, Paddington, £1,000. The bequests are for the endowment of a bed in each hospital, to be called the "W. and S. Roper Bed." Subject to the payment of a number of other public and private legacies, the testatrix left the residue of her estate to the following institutions: The North London Hospital for Consumption, Charing Cross Hospital, Victoria Hospital for Children, Middlesex Hospital, Guy's Hospital, St. Mary's Hospital, Paddington, the Hampstead Hospital and Nursing Institution.

At the annual meeting of the Royal National Sanatorium for Consumption, Bournemouth, the annual report of the committee of management, which was adopted, stated that during the past year 481 patients received the benefits of the charity, and that the results of the open-air treatment continued to be most satisfactory in the great majority of cases. The fact that the patients were received from 36 different counties of England, in addition to those who came from Wales, Scotland, and Ireland, establishes the claim of the sanatorium to be a national institution. A large proportion of the patients came from London. The average cost of each patient per week was £1 2s. 7d. This the committee considered would compare most favourably with any other institution of its kind, but they were anxious to state that, while exercising the strictest economy in administration, they had always insisted that everything should be done to ensure the comfort and proper care of the patients, especially with regard to the quality, quantity, and variety of the food provided. There was a considerable deficiency in the income for the year and in consequence the debt increased to £751. During the past year many improvements have been carried out, including the installation of new heating arrangements throughout the building, new domestic hot-water supply and gas-cooking appliances. Lord Wimborne, Mr. G. J. Fenwick, and Colonel Lewin, retiring by rotation, were re-elected members of the committee of management.

